# An Ornithomimid (Dinosauria) Bonebed from the Late Cretaceous of Alberta, with Implications for the Behavior, Classification, and Stratigraphy of North American Ornithomimids

**DOI:** 10.1371/journal.pone.0058853

**Published:** 2013-03-12

**Authors:** Thomas M. Cullen, Michael J. Ryan, Claudia Schröder-Adams, Philip J. Currie, Yoshitsugu Kobayashi

**Affiliations:** 1 Department of Earth Sciences, Carleton University, Ottawa, Ontario, Canada; 2 Department of Vertebrate Paleontology, Cleveland Museum of Natural History, Cleveland, Ohio, United States of America; 3 Department of Biological Sciences, University of Alberta, Edmonton, Alberta, Canada; 4 Hokkaido University Museum, Hokkaido University, Sapporo, Hokkaido, Japan; Ludwig-Maximilians-Universität München, Germany

## Abstract

Bonebeds can provide a wealth of anatomical, taphonomic, and ontogenetic information about the specimens preserved within them, and can provide evidence for inferred behavior. The material described here represents the first known bonebed of ornithomimids in North America, and the fourth record of an ornithomimosaur bonebed in the world. Partial skeletons representing three individuals are preserved in this assemblage, each comprising primarily portions of the posterior postcrania (pelvis, hind limbs and tail). All three individuals are morphologically similar, although one is larger in overall size. Given the stratigraphic position of the site, and the morphology of the postcrania, the preserved material represents a taxon from the clade containing *Ornithomimus* and *Struthiomimus*. Pedal ungual morphology is examined and found to be too variable to be useful in distinguishing these species taxonomically. This site provides additional evidence of gregarious behavior in ornithomimids and the first probable record of that behavior in North American forms.

## Introduction

Ornithomimosaurs are a group of gracile theropods known mainly from the Cretaceous of Asia and North America [1]. Ornithomimids, a derived sub-group of the ornithomimosaurs, are represented by relatively few complete skeletons [1]. Isolated postcranial elements, especially phalanges, are, conversely, very common. Although bonebeds are common for ornithischians, especially ceratopsians and hadrosaurs, theropod bonebeds are rare [2], [3], [4], [5]. Examples from the Late Cretaceous include an *Albertosaurus* bonebed from the Horseshoe Canyon Formation of Alberta [6], [7], a *Daspletosaurus* bonebed from the Two Medicine Formation of Montana [8], and a *Mapusaurus* bonebed from the Rio Limay Formation of Argentina [9]. Only two ornithomimid bonebeds have been reported, both from China [10], [11]. Additionally, a more basal ornithomimosaur bonebed has recently been reported from the Early Cretaceous of France [12].

**Figure 1:**
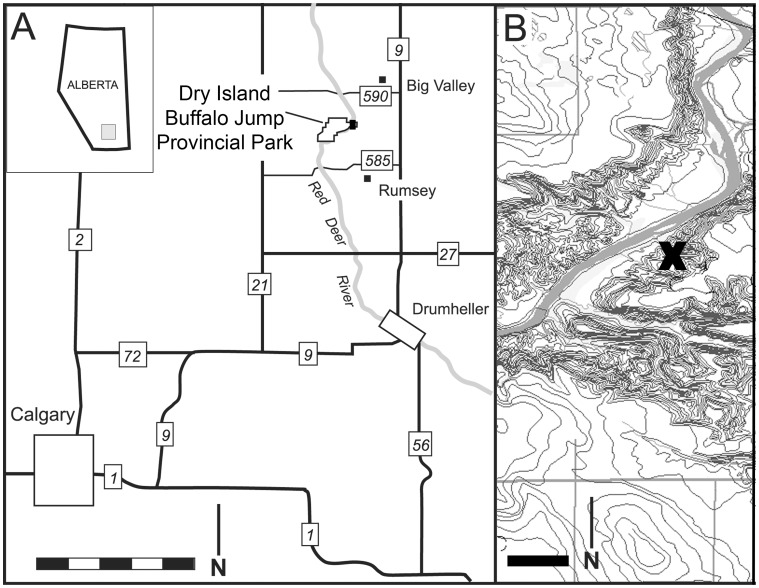
Geographic location of bonebed locality. **A**: Southern Alberta and Dry Island Buffalo Jump Provincial Park. Scale bar equals 50 km. Modified with permission from Eberth and Currie [7]. **B**: Dry Island Buffalo Jump Provincial Park with bonebed locality site indicated by X. Scale bars equal 1 km.

In 1926, C. M. Sternberg collected three partial ornithomimid skeletons (CMN [Canadian Museum of Nature, Ottawa, Canada] 12068, CMN 12069 and CMN 12070) from the east side of the Red Deer River (Figure 1) in what is now Dry Island Buffalo Jump Provincial Park. Here we provide the first description of these specimens. All three specimens lack skulls and forelimbs, making exact taxonomic determination difficult; they are here referred to Ornithomimidae due to the elongate metatarsus, relative straightness of the pedal unguals, and the loss of the first pedal digit [1], [13]. The locality represents the first ornithomimid bonebed known outside of Asia.

### Locality

C. M. Sternberg (unpublished field notes, 1926) gave the locality as being in the northeast quarter of section 28, township 34, range 21, approximately 4 miles northwest of Scollard, Alberta (Figure 1). The material was excavated 55 m above the Red Deer River, in what Sternberg described as “a clayie sand with some ironstone surrounding No. 7 [CMN 12070]”. This corresponds with the non-coaly interval of what is now referred to as the Tolman Member (previously Unit 4) of the lower Maastrichtian (70–68 Ma) Horseshoe Canyon Formation [14], [15].

The original quarry locality has not been relocated with absolute certainty. In 2005, P. Currie determined that the original quarry had probably been located on the edge of a small outcrop (51.9477°,–112.9190°) that has now been lost through erosion. This agrees with Sternberg’s unpublished field notes from 1926 where he noted that the ironstone around CMN 12070 had been almost completely eroded before discovery. He reported that all three specimens were found together, with nothing else but a few bone fragments and a single carnivorous dinosaur tooth associated with them. He interpreted the state and orientation of the specimens in the sediment as indicating original deposition after washing in from the northeast, and that the scattering of some ribs and phalanges was due to water action before burial and not scavenging. Unfortunately, a precise locality map showing the orientation of the specimens relative to one another and the deposit was not made during collection, nor did Sternberg make detailed notes on the taphonomy of the site.

## Results

### Description – General Comments

After their collection in 1926, the field jackets were opened in 1969 and partially prepared at the Canadian Museum of Nature. Additional minor preparation was carried out in 2009–2010 by the first author. Sternberg’s notes mention more material than currently exists, including a nearly complete vertebral column for CMN 12068. The museum preparation records do not match Sternberg’s list of elements found in the quarry, so some of the material was either not collected, or was lost prior to, or during, preparation in 1969. Most of the material remains within half jackets, and only their exposed surfaces, or stratigraphically upper surfaces, are prepared. Russell [13] briefly mentioned these specimens in his review of Canadian ornithomimid material, in which he assigned them to *Dromiceiomimus brevitertius* (now considered as a synonym of *Ornithomimus edmontonicus* [16], [17]), primarily on the basis of relative limb proportions. Two of the specimens are of similar size, with CMN 12068 representing an individual that is approximately 6–8% larger (based on femur length) than the others (see Table S1). The size of the larger individual (CMN 12068) is consistent with what is thought to represent adult to near-adult size in other similarly stratigraphically aged North American ornithomimids [1], [13]. Many of the elements from each skeleton are incomplete or in poor condition. Detailed measurements are listed in Table S1.

### CMN 12068

**Figure 2:**
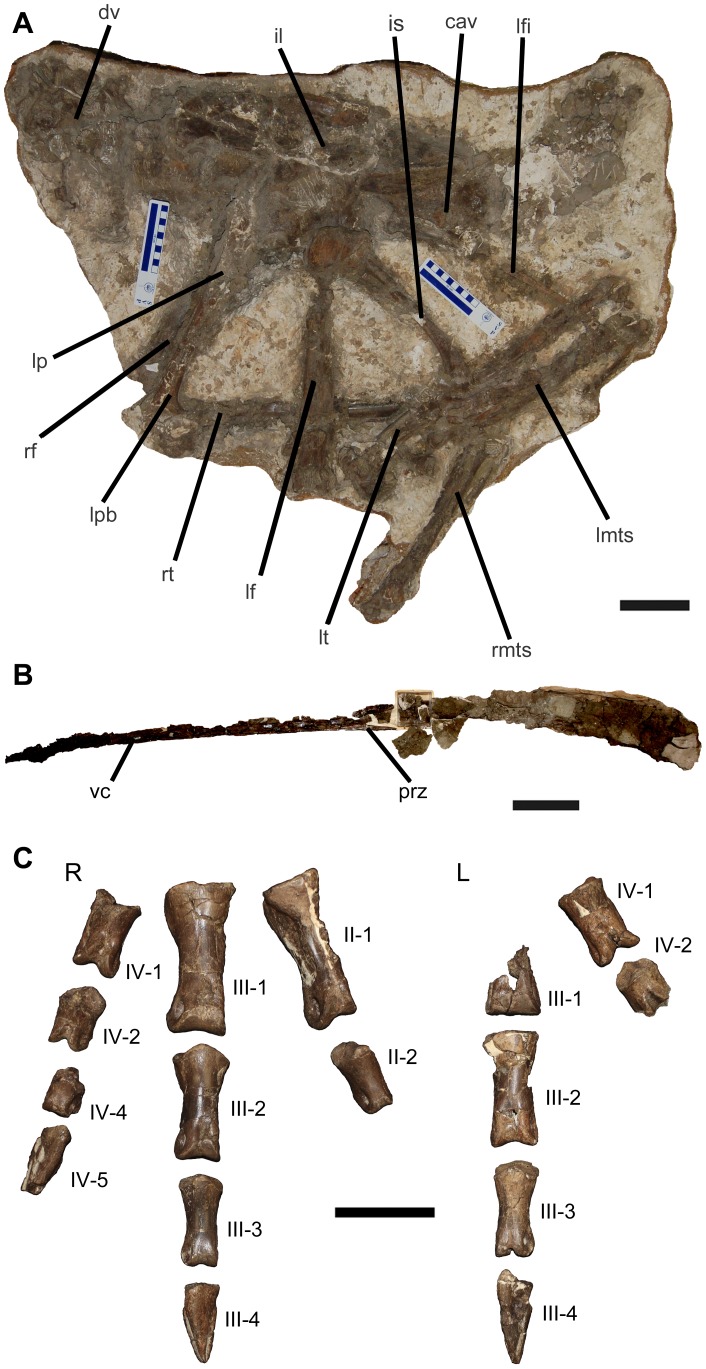
Material associated with CMN 12068. **A**: Large section of body showing majority of preserved material in left lateral view. **B**: Right lateral view of caudal vertebral section (ventral at top). **C**: Dorsal view of right and left pedes, with numbered phalanges. **Abbreviations**: **cav**, caudal vertebra; **dv**, dorsal vertebra; **il**, ilium; **is**, ischium; **lf**, left femur; **lfi**, left fibula; **lmts**, left metatarsals; **lp**, left pubis; **lpb**, left pubic boot; **lt**, left tibia; **prz**, prezygapophyseal bar; **rf**, right femur; **rmts**, right metatarsals; **rt**, right tibia; **vc**, vertebral centrum. Scale bars equal 10 cm.

CMN 12068 (Figure 2) (Table S1) is the largest, and most complete of the three skeletons. Although Sternberg described the skeleton as being mostly complete, nothing is now preserved in the CMN collection anterior to the 11th dorsal vertebra, nor was anything from this part of the body mentioned by Russell [13] in his review of ornithomimosaurs. The preserved elements of CMN 12068 are mostly articulated, but some were displaced up to several centimeters before burial. The appendicular elements (femora, tibiae, fibula, metatarsals, and pedal phalanges) make up the majority of the preserved material, with some axial material (partial sacrum, fragmentary dorsal vertebra, nearly complete though fragmentary caudal series) also preserved.

A number of characters found in this specimen allow it to be identified as belonging to Ornithomimidae. The ischium is curved anteroventrally, and the brevis fossa is well developed along the post-acetabular blade [16], [18]. The pubic shaft is nearly straight and the pubic boot is ventrally expanded [11], [18], [19]. The distal end of the pubic shaft is straight, with a large acute angle between the shaft and boot [16]. Additionally, the shaft of metatarsal III expands medially creating a ‘bulge’, and the proximal end of the shaft is covered by metatarsals II and IV, forming an ‘arctometatarsalian’ condition [1], [19]. This last character is potentially problematic if used as diagnostic of ornithomimids, particularly in combination with the brevis fossa, as it is also present in tyrannosaurs [20]. However, the lack of diagnostic tyrannosaur characters such as a vertical ridge on the lateral ilium surface precludes ornithomimids, such as this specimen, from tyrannosaur affinities [16].

The pedal phalanges are the best-preserved elements for this specimen, although neither foot is complete. Diagnostic of ornithomimids, the first pedal digit is absent, and phalanx II-2 is less than 60% the length of II-1 [16]. As in other ornithomimids, digit III is the longest, whereas digit IV is the shortest. The proximal articular surfaces of the proximal phalanges (II-1, III-1, IV-1) are shallowly concave and undivided. Phalanx IV-1 has some unusual pitting (possibly pathological) on the proximal articular surface. The proximal articular surfaces of more distal elements of digits II and IV are ginglymoid; distal articular surfaces are convex and grooved dorsoventrally. The unguals are straight in lateral view, and flat ventrally with weak flexor tubercles, again diagnostic of ornithomimids [16], [17], [21]. The ungual of digit III (III-4) is directed anteriorly, and is flat ventrally with a weak proximal concavity. It has lateral and medial grooves on the dorsal surface extending from the proximal articulation to the distal tip. Ungual III-4 is well preserved but has some abrasion on the proximodorsal surface.

### CMN 12069

**Figure 3:**
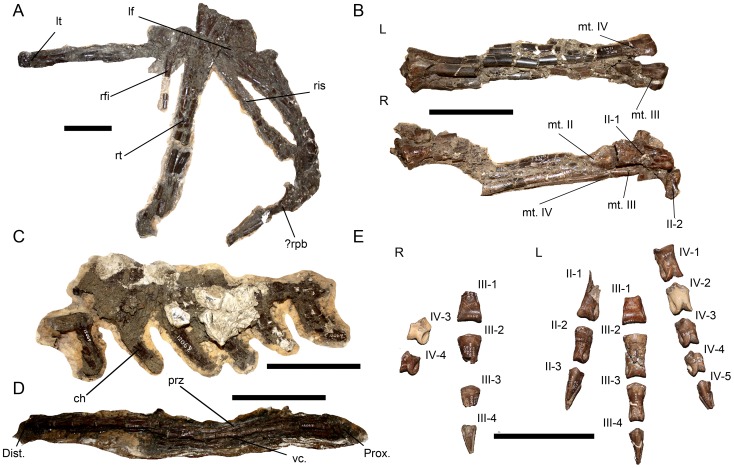
Material associated with CMN 12069. **A**: Appendicular elements in lateral view. **B**: Left and right metatarsals, and proximal phalanges, in dorsolateral view. **C**: Proximal section of caudal series in left lateral view. **D**: Distal section of caudal series in right lateral view. **E**: Dorsal view of right and left pedes, with numbered phalanges. **Abbreviations**: **ch**, chevron; **lf**, left femur; **lt**, left tibia; **mt. II–IV**, metatarsals II–IV; **prz**, prezygapophyseal bar; **rfi**, right fibula; **ris**, right ischium; **rt**, right tibia; **?rpb**, possible right pubic boot; **vc**, vertebral centrum; **II-1/II-2**, phalanx elements II-1/II-2. Scale bars equal 10 cm.

CMN 12069 is less complete than CMN 12068, but is generally the best preserved of the three individuals with regards to vertebrae or pedes, with a number of appendicular elements also preserved (Figure 3) (Table S1).

This material is heavily fractured and weathered, with the exception of a section of distal caudal vertebrae (numbers 23–32), making accurate identification and description of individual elements difficult. The majority of the fracturing is orthogonal, with some minor offsetting, while the surface weathering appears consistent with post-burial exposure. In the section of caudal vertebrae, the prezygapophyses of each vertebra overlap 40–80% of the preceding centrum. The centra are taller than wide in lateral view, similar to those of other ornithomimids [13].

The left and right metatarsals are fractured, and in the case of the right metatarsals articulate with some proximal phalanges. The right pes is incomplete, but well preserved, and the left pes is complete, in that all elements are present, although phalanges II-1, III-1, and III-2 are badly fragmented. The pedal elements of CMN 12069 are generally smaller than those of CMN 12068, although otherwise similar.

### CMN 12070

**Figure 4:**
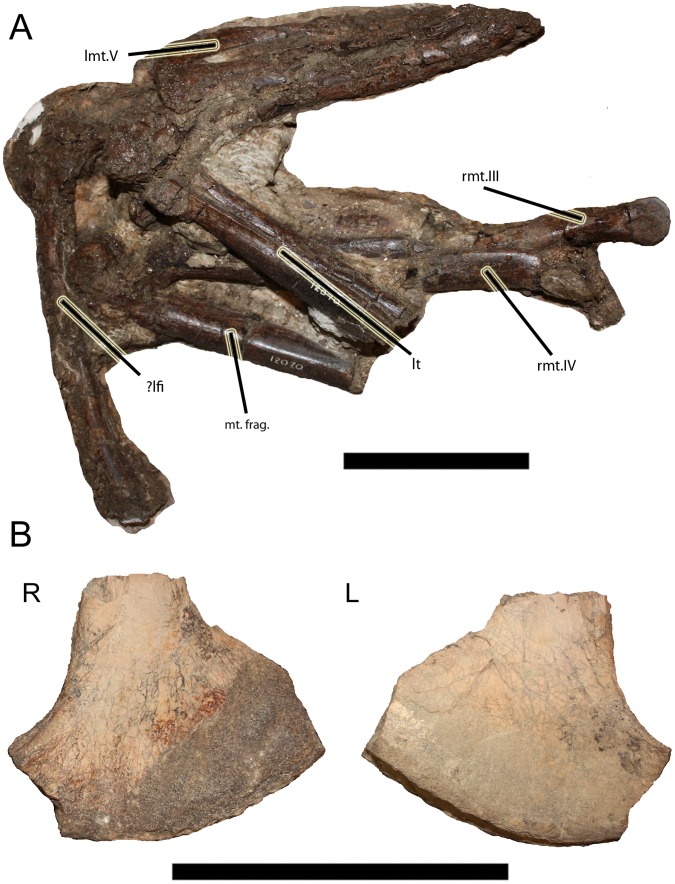
Material associated with CMN 12070. **A**: Fragmentary lower hind limbs, primarily fragmentary metatarsals, in lateral views. **B**: Right and left lateral views of pubic boot. **Abbreviations**: **lmt.V**, left metatarsal V; **lt**, left tibia; **mt.frag**, metatarsal fragment; **rmt.III**, right metatarsal III; **rmt.IV**, right metatarsal IV; **?lfi**, possible left fibula. Scale bars equal 10 cm.

CMN 12070 (Figure 4) (Table S1) is the least complete and most poorly preserved specimen. It includes a series of four proximal caudal vertebrae and associated chevrons, an isolated pubic boot, fragmentary metatarsals, and two pedal phalanges (both III-2). The pubic boot is of note diagnostically, as it is expanded anteriorly, an ornithomimid character [16].

### Taphonomy

These specimens have undergone very little abrasion, showing Stage 0 or 1 conditions, suggesting they are both associated in time and originate from a single event [2], [22], [23]. This material shows a moderate to high degree of compressional fracturing, consistent with deposition within fine-grained sand to silt [2]. Longitudinal fracturing is present on much of the material as well, suggesting periods of prolonged post-depositional weathering [2]. This is further supported by nearly all of the material showing Stage 2 weathering, with some elements ranging from Stage 0 or 1 (most pedal phalanges and some metatarsal material) to Stage 3 (segments of CMN 12069 long bone material and CMN 12068 caudal vertebrae [2], [22]). No scratches or bite marks appear to be present on the bone, and the only other theropod material present is a single small shed tyrannosaurid tooth, identified as such due to the presence of a lingually ‘twisted’ tooth tip [24]. This suggests that the material was not highly scavenged, nor was it moved into position by predators [2], [23]. In summary, most of the material at this site is preserved in moderate to poor condition, but this appears to be due in large part to post-depositional fracturing and weathering, with little damage due to abrasion or scavenger action.

### Phylogenetic and Principal Component Analyses

**Figure 5:**
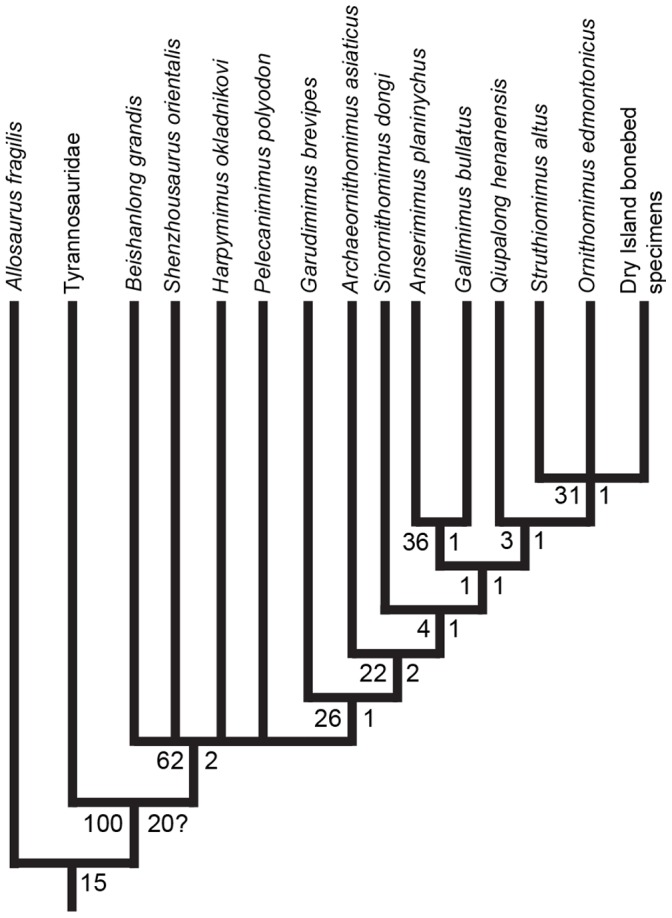
Results of phylogenetic analysis of ornithomimosaur taxa. Results based upon analysis of a modified character/taxon matrix of Xu et al. [16]. Strict consensus of 5 most parsimonious trees (CI = 0.67, RI = 0.75). Fourteen of 31 post-cranial characters could be coded for the Dry Island bonebed specimens. Bootstrap and Bremer support values indicated at left and right, respectively, of each branch point.

The phylogenetic analysis of the Dry Island bonebed ornithomimid specimens generated 5 most parsimonious trees each of 75 steps length (consistency index = 0.667, retention index = 0.747). The strict consensus of those five trees is shown in Figure 5, with bootstrap (100,000 replicates) and Bremer support values indicated. *Ornithomimus*+*Struthiomimus*+Dry Island specimens form an unresolved polytomy nested within Ornithomimidae, sister clade to *Qiupalong henanensis*.

A principal component analysis (PCA) was performed in an attempt to further classify the Dry Island ornithomimid bonebed specimens beyond the polytomy recovered by the phylogenetic analysis. The results (Table S3, Figure S1, Figure S2) showed no significant statistical differences between any of the included specimens.

## Discussion

### Taxonomic Identification of the Dry Island Ornithomimid Specimens

While the characters presented in the description of this material confirms that the Dry Island specimens are placed within Ornithomimidae, narrower taxonomic determination of the skeletons is difficult given that the skulls, containing most of the diagnostic characters, are missing from each of the three specimens [1], [16]. The postcrania (excluding the distal forelimb which is not present in any of the specimens) are of limited taxonomic utility in distinguishing between individual North American ornithomimid species [1], [16], [21]. The posterior caudal vertebrae are too fragmented to identify ridge and groove articulations [25]. In addition, previous studies have shown that relative limb proportions produce inconclusive results [26], [27]. The original descriptions and early studies of North American ornithomimids contained a number of additional postcranial characters, but many of these are no longer widely accepted as valid in distinguishing taxa [1], [13], [16], [21], [28], [29], [30], [31].

The phylogenetic analysis of the bonebed specimens grouped them in a polytomy with *O. edmontonicus* and *S. altus*, although given the low bootstrap and Bremer support values throughout the phylogeny this result can be said to be poorly supported. However, the purpose of the study is not to resolve ornithomimid phylogeny, but to report on the first North American occurrence of an ornithomimid bonebed. The poor support values throughout the clade underscore the need for a taxonomic reassessment of North American forms, and additional work in understanding all ornithomimosaurs. As noted, although the phylogenetic analysis allowed some narrowing of the classification, the reported material does not contain characters that can presently be used to differentiate between the two ornithomimid taxa currently known from the Horseshoe Canyon Formation, *Struthiomimus* and *Ornithomimus*. Although our material could be scored for 14 of 31 postcranial characters used in recent analyses (e.g. [16]), 13 of these are shared between *Struthiomimus* and *Ornithomimus*, as well as many other ornithomimids. The remaining character (convex and expanded ventral border of the pubic boot) unites both taxa within the clade *Struthiomimus*+*Ornithomimus*, and therefore cannot be used to differentiate between them [16], [21]. Consequently, the phylogenetic analysis resolved the Dry Island bonebed material in a polytomy with *Struthiomimus* and *Ornithomimus*. Longrich [17] recently advocated using pedal ungual characters (e.g. weakly vs. moderately curved claw; narrow vs. relatively broad in ventral view; sharp vs. round ventrolateral edges; poorly developed vs. reduced but distinct proximodorsal process) to differentiate between Albertan ornithomimids (specifically *O. edmontonicus* and *S. altus*). Our re-examination of these characters in CMN 8632 (*O. edmontonicus*), CMN 930 (*S. altus*), and CMN 12069 (Figure S3) could not duplicate his results, because we found these characters to be variably present within the clade of *Struthiomimus*+*Ornithomimus*. As a specific example, in CMN 12069 pedal ungual II-3 was found to possess a poorly developed proximodorsal process and an elongate claw, and to be narrow in ventral view, moderately curved, and with rounded ventrolateral edges. The latter two characters are considered diagnostic for *S. altus*, while the preceding three characters are diagnostic of *Ornithomimus* sp. Ungual III-4 displayed a poorly developed proximodorsal process, a weakly curved claw, narrow in ventral view (all *Ornithomimus* sp. characters), and with possibly rounded ventrolateral edges (*S. altus* character). Ungual IV-5 showed a weak to moderately curved claw, which was relatively broad in ventral view, with sharp ventrolateral edges, and a reduced but distinct proximodorsal process (again a combination of characters purported to be diagnostic of either *S. altus* or *Ornithomimus* sp.). As applying these characters to a single individual results in different pedal digits being referred to different taxa, it can be said that the pedal unguals appear to be too variable for these characters to be useful in distinguishing North American ornithomimids. It is possible that the characters identified by Longrich are not entirely without merit, but they are currently too subjective to be useful in distinguishing unguals with as much apparent variation as seen in these two taxa. Additionally, the PCA and CVA performed on pedal phalanx shape produced similarly inconclusive results (Figures S1 & S2).

**Figure 6:**
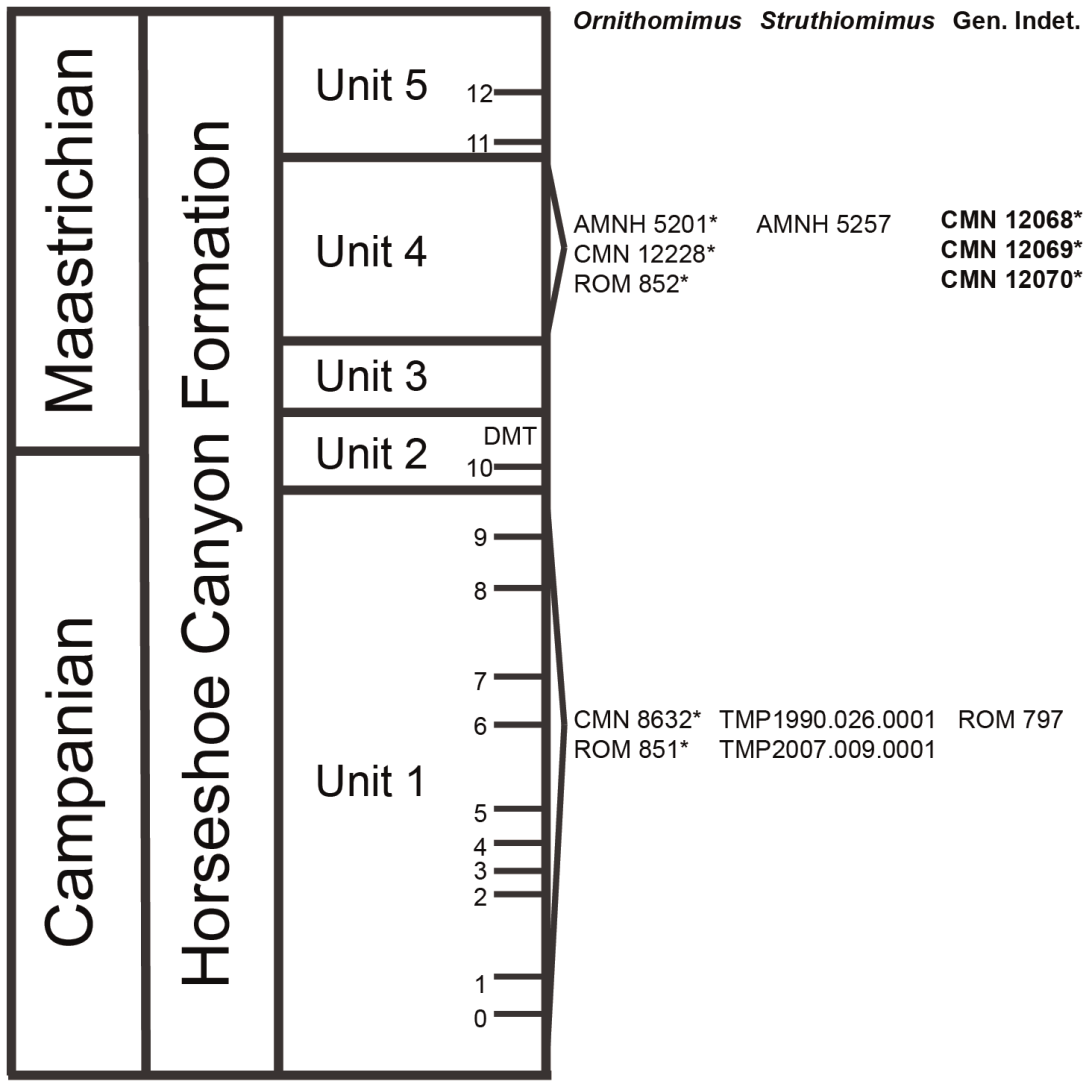
Stratigraphic occurrences of ornithomimids within the Horseshoe Canyon Formation. Asterisk (*) indicates specimens referred by Russell [13] to *Dromiceiomimus brevitertius*. Specimens described in this study are marked in bold. Numbers 0–12 represent coal marker beds within the Horseshoe Canyon Formation. Abbreviations: **CMN**, Canadian Museum of Nature; **DMT**, Drumheller Marine Transgression; **ROM**, Royal Ontario Museum; **RTMP**, Royal Tyrrell Museum of Palaeontology.

The stratigraphic position of the specimens can also be of assistance in the taxonomic identification of the bonebed material (Figure 6). Known ornithomimid material from the section of the Horseshoe Canyon Formation containing the bonebed (Tolman Member/Unit 4, above the Drumheller Marine Transgression) almost exclusively represents *Ornithomimus edmontonicus* (six skeletons in various states of completeness), with the exception of one specimen of *Struthiomimus altus* [1], [13], [21]. This further supports the placement of the bonebed specimens within the clade containing *Ornithomimus* and *Struthiomimus* [11], [16]. Although only one specimen of *S. altus* is currently known from this unit, it contains diagnostic forelimb material that makes misidentification unlikely [13]. In combination with the inconclusive phylogenetic and PCA results, we conclude that an exact species determination for the Dry Island bonebed material cannot be confirmed at this time.

### Taphonomy and Behavioral Implications

The association of the incomplete ornithomimid skeletons CMN 12068, 12069, and 12070 in one quarry is interpreted as the first known ornithomimid bonebed (sensu Eberth et al. [6]) from North America, the third ornithomimid bonebed occurrence worldwide, and the fourth occurrence of an ornithomimosaur bonebed, when the bonebed of southwestern France is included [10], [11], [12]. The partially articulated state of these skeletons and taphonomic conditions of the site suggests that the specimens, if transported, were moved only a relatively short distance [30]. Alternatively, these individuals may have died in situ and some elements (or even other complete carcasses) could have washed away before final burial, or while exposed prior to collection, a position supported by the evidence of prolonged weathering seen on the bones [2]. Also, as implied by Sternberg in his 1926 field notes, additional skeletons may have been present at the time of deposition, but were subsequently lost by recent erosion. This could explain why the preserved part of each skeleton is articulated, whereas the remainder of the skeleton and disarticulated bones were not found.

Two additional ornithomimid bonebeds have been found in Asia [10], [11]. The first described occurrence was at Iren Dabasu, in the People’s Republic of China [1], [10], [32] and contains the remains of *Archaeornithomimus*. This bonebed is composed of the disarticulated skeletons of almost 30 individuals spread across three quarries approximately 30 m from each other [32]. The scattered occurrence implies that the material either travelled some distance before deposition, or that it was disturbed in situ through scavenging. The locality is interpreted as a braided fluvial system separated by broad, shallow channels that periodically spilt onto the floodplains in a semi-arid terrestrial environment with some coastal influences [10]. The second bonebed containing at least 34 articulated skeletons of *Sinornithomimus dongi* is in Inner Mongolia at a locality called Ulan Suhai, People’s Republic of China [11], [33]. Given that the material is uniformly well preserved, and that there is no evidence of scavenging, the bonebed is interpreted as a mass mortality event in which the carcasses were quickly buried [11]. Juvenile- and sub-adult-sized material is known from this site, with 22 (67%) of the individuals considered to be juveniles based on small body size and bone histology [11], [33]. Given the upright posture of individuals from this site and the presence of fine muds and silts, it has been suggested that this site represents a lacustrine mud-trap, which in turn favored fossil preservation [33]. In contrast, the Dry Island bonebed is interpreted as having been deposited in an alluvial setting, which could account for the poorer preservation conditions and lower density of individuals.

This study provides the first description of an ornithomimid bonebed known from North America, and one of only three such sites worldwide. Although the taxonomic identity of the specimens could not be determined beyond the clade containing *Ornithomimus* and *Struthiomimus*, it provides further evidence of gregarious behavior in ornithomimids, and highlights some of the outstanding problems within ornithomimid classification.

## Materials and Methods

### Measurements

All individual elements contained within the bonebed specimens were measured using digital calipers, with each measurement taken three times and averaged. The list of individual measurements can be found in Table S1.

### Analytical Methods

Specimens were prepared from their plaster jackets using standard paleontological preparation techniques. Taphonomic condition scales used in this study follow Ryan et al. [2]. To assess the relationship of the Dry Island bone bed specimens described here to other ornithomimid taxa we scored them for a cladistic data matrix (Table S2), modified from Xu et al. [16]. The ‘Dry Island bonebed specimens’ were scored as a combination of CMN 12068, 12069, and 12070, which were coded the same for each specimen and therefore analyzed as a single operational taxonomic unit. We performed a phylogenetic analysis using TNT [34], with a traditional Wagner search with 1000 replicates using tree bisection reconnection branch swapping. A principal component analysis (PCA) was performed in an attempt to further classify the Dry Island ornithomimid bonebed specimens beyond the polytomy resolved in the phylogenetic analysis. The PCA was performed using the lengths, proximal and distal heights, and proximal and distal widths of each pedal phalanx from the Dry Island bonebed specimens and several articulated specimens of *Ornithomimus* and *Struthiomimus* (CMN 8632 [*O. edmontonicus*], CMN 930 [*S. altus*], ROM [Royal Ontario Museum, Toronto, Canada] 852 [“*O. edmontonicus*”], ROM 851 [*O. edmontonicus*], ROM 797 [“*O. edmontonicus*”]), ROM 1790 [“*S. altus*”]). Left/right averages were produced for each specimen, and unshared material was removed. The full list of measurements can be found in Table S3. A canonical variate analysis (CVA) was then performed, on the measurements identified in the PCA as most affecting the variation, to test the significance of clustering in the morphospace. The PCA and CVA were performed on these data using PAST [35]. Resultant plots can be found in Figures S1 and S2, respectively. No permits were required for the described study, which complied with all relevant regulations.

## Supporting Information

Figure S1 **Principal component analysis results.**
(TIF)

Figure S2 **Canonical variate analysis results.**
(TIF)

Figure S3 **Pedal unguals of CMN 12069, in dorsal, ventral, and lateral views.** **A**, digit II; **B**, digit III; **C**, digit IV.
(TIF)

Table S1 **Skeletal measurements of CMN 12068, 12069, and 12070.** Asterisk (*) indicates fragmentary, distorted or incomplete remains. Dash (-) indicates elements that are not present.
(XLS)

Table S2 **Data matrix used in phylogenetic analysis.**
(XLS)

Table S3 **Pedal phalanx measurements used in principal component analysis.** Unshared measurements removed. **Abbreviations**: **DH**, distal width; **DW**, distal width; **L**, length; **PH**, proximal height; **PW**, proximal width.
(XLS)
