# Supplementary figures and images for: An Ornithomimid (Dinosauria) Bonebed from the Late Cretaceous of Alberta, with Implications for the Behavior, Classification, and Stratigraphy of North American Ornithomimids
Source: PLoS One. 2013 Mar 12;8(3):e58853. doi: 10.1371/journal.pone.0058853 (PMC3595220; doi:10.1371/journal.pone.0058853)

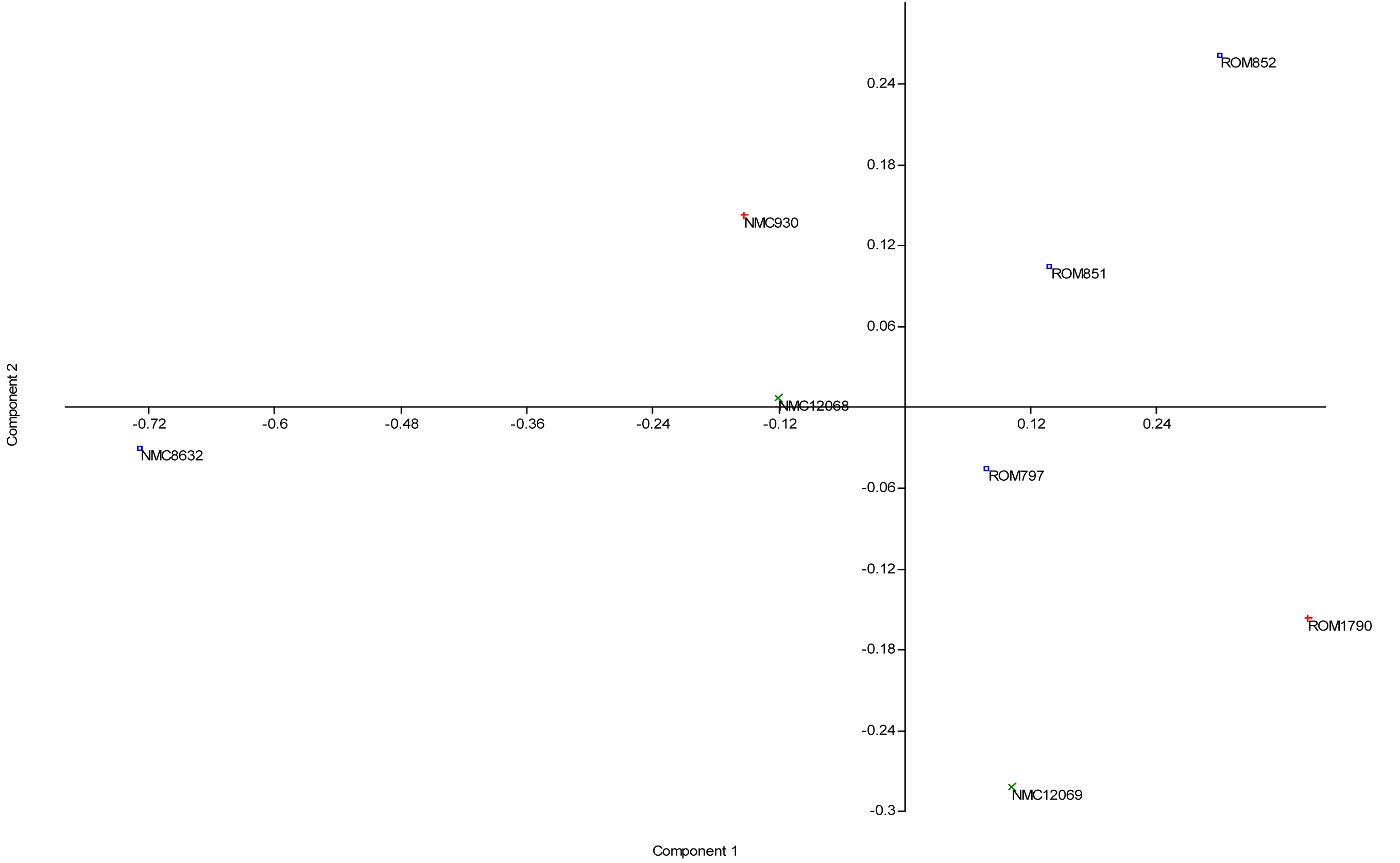

Supplement: Figure S1 — Principal component analysis results. (TIF) [file pone.0058853.s001.tif]

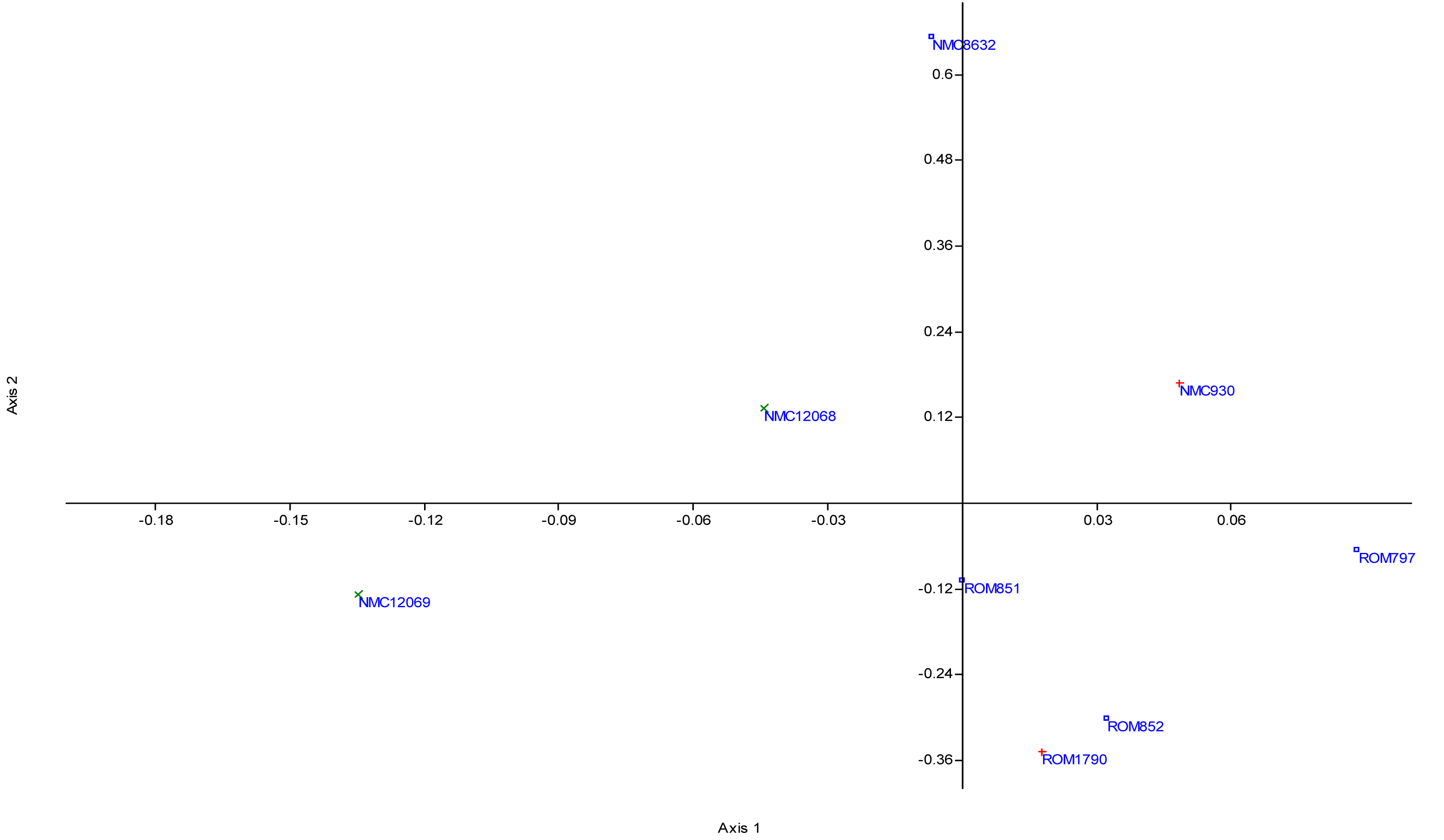

Supplement: Figure S2 — Canonical variate analysis results. (TIF) [file pone.0058853.s002.tif]

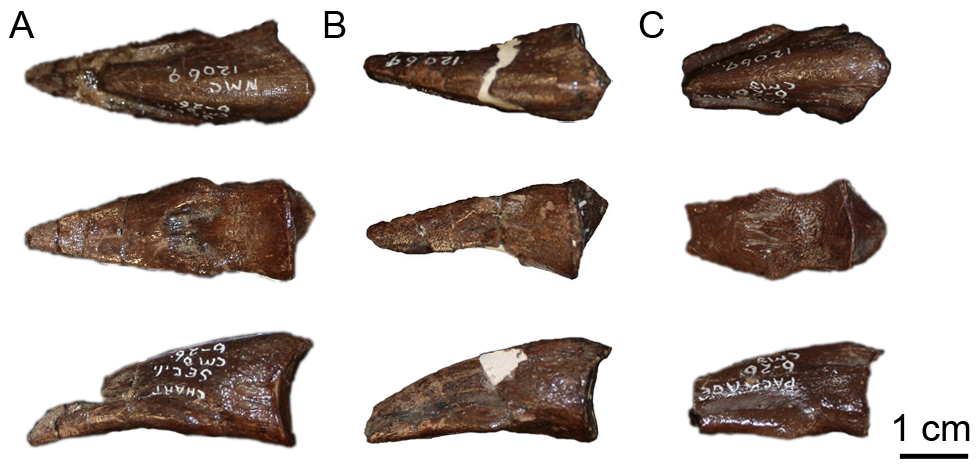

Supplement: Figure S3 — Pedal unguals of CMN 12069, in dorsal, ventral, and lateral views. A, digit II; B, digit III; C, digit IV. (TIF) [file pone.0058853.s003.tif]
